# Initial Emergency Department Diagnosis and Management of Adult Patients with Severe Sepsis and Septic Shock

**DOI:** 10.1186/1757-7241-20-41

**Published:** 2012-06-27

**Authors:** Sarah M Perman, Munish Goyal, David F Gaieski

**Affiliations:** 1 Department of Emergency Medicine, Perelman School of Medicine at the University of Pennsylvania, Pennsylvania, USA; 2 Department of Emergency Medicine, MedStar Washington Hospital Center; Georgetown University School of Medicine, Georgetown, USA; 3 Center for Resuscitation Science, Department of Emergency Medicine, Perelman School of Medicine at the University of Pennsylvania, 3400 Spruce Street, Philadelphia, PA 19104, USA

**Keywords:** Sepsis, Septic shock, Early-goal directed therapy, Resuscitation

## Abstract

Severe sepsis is a medical emergency affecting up to 18 million individuals world wide, with an annual incidence of 750,000 in North America alone. Mortality ranges between 28-50% of those individuals stricken by severe sepsis. Sepsis is a time critical illness, requiring early identification and prompt intervention in order to improve outcomes. This observation has led to increased awareness and education in the field of Emergency Medicine; it has also led to the implementation of critical interventions early in the course of patient management, specifically Early-Goal Directed Therapy, and rapid administration of appropriate antimicrobials. This review begins with a brief summary of the pathophysiology of sepsis, and then addresses the fundamental clinical aspects of ED identification and resuscitation of the septic patient.

## Background and Introduction

Severe sepsis and septic shock are medical emergencies which affect 18 million individuals per year world wide. [1,2] This rate may actually represent an underestimation on a global assessment, as some developing countries lack the advanced microbiology services necessary to quantify the extent of devastation secondary to infection. In North America 750,000 individuals with severe sepsis are hospitalized annually, and approximately 215,000 of those patient encounters result in death. [2] Internationally, 1400 individuals die each day with the care of septic patients costing European countries 7.6 billion euros annually. [2,3] On average, $16.7 billion US dollars are spent to care for the severely septic patient each year, with costs projected to rise by 1.5% per year. [2] Specifically in US emergency departments (EDs), suspected severe sepsis accounts for approximately 500,000 ED visits annually [4] and investigators have determined that in patients with septic shock, the rate of mortality increases by 7.6% for each hour that effective antimicrobials are delayed. [5] Therefore care of the severely septic patient is of great interest to the emergency physician and other care providers who are responsible for the initial management of this patient population.

Optimization of ED management of the septic patient is a priority, as North American studies have shown that two-thirds of septic patients enter the health system via the ED, but also due to the acuity of illness at initial presentation and the potential to alter patient outcome with emergent interventions. Historically, invasive management and aggressive resuscitation of the septic patient occurred in the intensive care unit (ICU); however, with further investigation, sepsis is now recognized as an overwhelmingly time critical disease, requiring early initiation of care. In 2001, Rivers et al. published a landmark paper “Early goal-directed therapy in the treatment of severe sepsis and septic shock,” describing a trial in which 263 patients were randomized to either standard care or early goal-directed therapy (EGDT), an aggressive algorithmic approach to resuscitation initiated at the earliest stage of critical infection. [6] By applying EGDT, the authors realized a 16% absolute reduction in mortality. [6] This randomized control trial laid the foundation for significant advances in the care of the septic patient and further solidified the role of the emergency care provider in identification of the disease and initiation of treatment. Additionally, hospital overcrowding has become a public health dilemma in many countries and optimizing sepsis care within the ED has become a high priority as patients wait ever longer for ICU and ward beds. [4] In a recent Canadian study, Green and McIntyre determined that critically ill patients awaiting ICU admission had a median length of stay of 4.9 hours. [7].

In 1999 the American College of Critical Care Medicine and the Society for Critical Care Medicine issued a “Practice parameter for hemodynamic support of sepsis in adult patients in sepsis” where experts made recommendations on fluid resuscitation, vasopressor therapy, inotropic therapy and hemodynamic endpoints that should be employed in the care of septic patients. [8] Shortly afterwards, The Surviving Sepsis Campaign (SSC), a consensus group of 55 international experts convened and published a group of guidelines for the care of the septic patient. The first SSC Guidelines were published in 2004; they were subsequently revised in 2008. The goals of the SSC were to present uniform definitions of severe sepsis and septic shock as well as to issue comprehensive, evidence-based guidelines on the optimal care of the septic patient. These guidelines were divided into an initial six-hour resuscitation bundle and a subsequent 6–24 hour bundle for longer-term management of the critically ill septic patient. [9,10] Despite published guidelines, the use of these therapies for the care of the septic patient remains low. [11,12] Studies have shown that implementation of bundled care as recommended by SSC may decrease mortality; however, only approximately 30% of patients received bundled care. [13].

### Pathophysiology

Sepsis is a disease process that is initiated by introduction of pathogens into the human host. Host cell recognition of these pathogens as “foreign” results in the release of the cytosolic nuclear factor, NF-κβ, which binds to the nucleus and triggers the production of various cytokines within the inflammatory cascade. [14] In addition, polymorphonuclear leukocytes become activated and express adhesion molecules resulting in margination or mobilization of these white blood cells to the source of infection. The sum of these processes is a pro-inflammatory response, which is modulated by an anti-inflammatory response mounted by the same host organism. The pro-inflammatory response, mediated by inflammatory markers including TNF-α, interferon γ, interleukin (IL)-1, IL-2, and IL-6, triggers the release of additional mediators, which results in characteristic clinical findings of inflammation including fever, tachycardia and tachypnea. However, investigators have found that as sepsis persists there is a shift toward an anti-inflammatory response mediated by IL-4 and IL-10. It is theorized that overwhelming sepsis occurs due to a dysregulation in the complex balance between pro-inflammatory mediators or the systemic inflammatory response (SIRS), and anti-inflammatory mediators, or the compensatory anti-inflammatory response (CARS), resulting in processes that directly damage endothelial, cardiovascular, hemodynamic and coagulation mechanisms. [15] The end result of these pro- and anti-inflammatory responses is cardiovascular compromise, apoptosis (cell death), irreversible organ dysfunction, suppression of the immune response, and death.

### Clinical features

Sepsis is defined as the presence of a source of infection and evidence of a systemic inflammatory response to that infection as measured by the existence of two or more SIRS criteria. Clinical features of sepsis include three of the SIRS criteria; a temperature greater than 100.4°F or less than 96.8°F (>38.0°C or < 36.0°C); tachycardia, a heart rate greater than 90 beats/minute; and tachypnea, a respiratory rate greater than 20 breaths/minute; the basic laboratory finding of sepsis is a white blood cell count greater than 12 thousand/mm^3^ or less than 4 thousand/mm^3^ or greater than 10% immature cells and constitutes the fourth SIRS criteria.

The above scenario, coupled with signs of end organ dysfunction due to microvascular compromise and poor perfusion, defines severe sepsis. De Backer et al. utilized an orthogonal polarization spectral imaging technique to establish that sublingual microcirculation in septic patients is impaired in comparison to healthy volunteers and non-septic critically ill patients. Additionally, the proportion of perfused small vessels was directly correlated with survival, where survivors had a higher rate of perfusion. [16] Poor perfusion of the brain may result in altered mental status; poor renal perfusion may lead to oliguria or anuria; poor cardiac perfusion may result in myocardial depression, decreased cardiac output, and hypotension or signs of heart failure; skin may be mottled; lung dysfunction may result in acute lung injury or acute respiratory distress syndrome.

Septic shock is a subset of severe sepsis characterized by hypotension unresponsive to fluid resuscitation. Sepsis-induced hypotension is defined as a systolic blood pressure (SBP) less than 90 mmHg or a reduction of greater than 40 mmHg from baseline. [3] Despite the significant end organ dysfunction found in severe sepsis, the actual mechanism for why patients die from sepsis is not clearly known.

### Early Identification and diagnostic criteria

**Figure 1:**
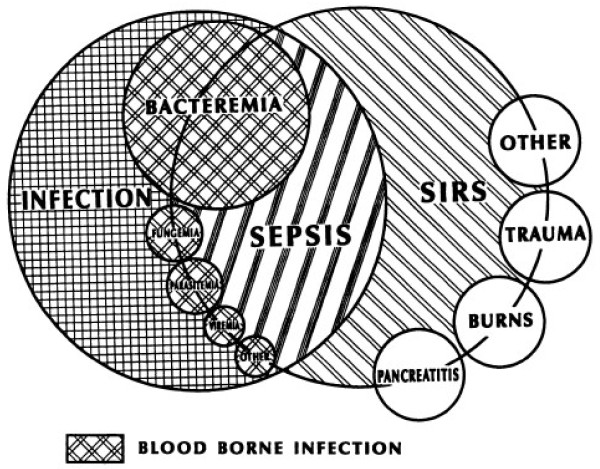
**The interrelationship between systemic inflammatory response syndrome (SIRS), sepsis, and Infection.** Used with the permission of *CHEST.*License number2835980252893.

The initial management of sepsis requires correct identification of septic patients by acute care providers. Emergency department triage systems are designed to classify patients by severity of illness, with an initial set of vital signs, chief complaint, and focused physical exam. During this first encounter with the health care delivery system, much information can be gleaned with respect to the presence or potential for evolution of severe sepsis and septic shock. It is critical to recognize that not all individuals who have SIRS criteria are septic, nor is it accurate to assume that patients without SIRS criteria are not septic. As Bone et al. discussed in the ACCP/SCCM consensus statement in 1992 there is much overlap in these initial hemodynamic alterations with disease entities such as burns, trauma, and pancreatitis and practitioners must render further clinical judgment in order to accurately diagnose the septic patient (Figure 1). [3] Comstedt et al. evaluated acutely ill individuals admitted to a Danish hospital and found that 35% of this cohort met SIRS criteria on admission and had an increased likelihood of infection (relative risk, 2.2) within two days of admission versus the non-SIRS cohort. The SIRS cohort was also found to have a 6.9 times greater risk of 28-day mortality than the non-SIRS cohort. [17] Similarly, in 2006 Shapiro et al. found that in 3102 adult patients with suspected infection, the presence of SIRS criteria alone had no prognostic value, but that identifying organ dysfunction yielded relevant prognostic information. [18] In total, 34% of patients with severe sepsis and 24% of patients with septic shock did not meet SIRS criteria during their ED stay. Therefore, SIRS criteria do not necessarily define the sepsis syndrome, and first line emergency care practitioners should perform a thorough physical exam and incorporate the results of additional studies in order to accurately define this cohort of patients.

Several mechanisms have been proposed to assist in the identification of patients with sepsis. Recent attention has focused on the topic of biomarkers, with serum lactate being the best studied. Whether a marker of poor perfusion, leading to increased lactate production, or an indicator of impaired clearance secondary to organ dysfunction, multiple studies have shown that elevation in serum lactate is an effective marker for risk stratification of severe sepsis patients in the ED. [19,20] In a recent study by Mikkelsen et al., the investigators demonstrated that elevations in serum lactate independent of clinically relevant hypotension were associated with increased mortality in patients who present to the ED with severe sepsis. [21] Taken together, these studies suggest that serum lactate may perform well as a screening test in the medical decision-making process regarding early ED management and disposition of the septic patient.

As early as 1964, Dr. Max Harry Weil proposed that serum lactate measured during critical illness correlated with adverse outcomes. Broder and Weil determined that in a cohort of patients with mixed critical illness, individuals with a lactate excess of at least 4 mmol/L had a mortality rate of 87%. [22] This finding was further validated by Aduen and colleagues in 1994 measuring lactate levels (not lactate excess) [23] and was subsequently used by Rivers et al. as an inclusion criterion for patients screened for the original EGDT trial. [6].

More recently, investigators have begun to address elevated serum lactate levels that are lower than the 4 mmol/L traditionally used to capture patients for protocolized resuscitation. The rationale behind this approach is to identify and treat severe sepsis patients earlier in their clinical course to halt the inflammatory cascade and reverse perfusion abnormalities before they progress further. Shapiro et al. analyzed 1,280 medical records of patients with sepsis who had a low lactate level (0–2.5 mmol/L) vs. intermediate lactate level (2.5-3.9 mmol/L) vs. high lactate (≥ 4.0 mmol/L) and determined that their mortality rates were 4.9%, 9.0% and 28.4% respectively. [20] Similarly, Mikkelsen et al. found that hemodynamically stable individuals with a lactate between 2.0-3.9 mmol/L had a mortality rate that was twice that of those with a lactate less than 2.0 mmol/L. [21] These recent findings are consistent with original research demonstrating that a lactate ≥ 4.0 mmol/L is concerning for end organ dysfunction and adverse outcomes; in addition, they suggest that patients with an intermediate lactate level are also at increased risk of adverse outcome relative to those with a normal lactate level.

Other techniques have been applied in order to determine illness severity of the septic patient in the ED. In one study, investigators examined the utility of quantifying the number of organ systems that were impaired as a prognostic factor. They reviewed the charts of 3,102 individuals who had been admitted from the ED with a diagnosis of sepsis. From this chart review, they determined that the mortality rate increased with each additional organ system that was impaired (increased adjusted 1-year mortality hazard ratio by 82%). [18].

Another approach to early identification of high risk ED patients with sepsis is to use illness severity scoring systems. For example, Shapiro et al. reviewed 3000 patient encounters in order to create a 9-part ED-specific scoring system titled the Mortality in Emergency Department Sepsis (MEDS) score. This instrument stratifies patients into mortality risk groups of very low, low, moderate, high, and very high. Mortality rates in these risk groups were 1.1%, 4.4%, 9.3%, 16% and 39% respectively. The area under the receiver operating characteristic (ROC) curve was 0.82 in the derivation set and 0.78 in the validation set. [24] Unfortunately, when Jones et al. attempted to validate the MEDS score, they found that it performed poorly within their cohort, with an area under the ROC curve of 0.61 (95% CI 0.50-0.72). [25].

Attempts have also been made to validate currently employed ICU scoring systems in the ED setting to risk stratify patients with possible sepsis. In the ICU setting, the Sequential Organ Failure Assessment (SOFA) score numerically quantifies organ impairment at 24-hour intervals. The SOFA score is a validated objective score that allows for assessment of the number of impaired organs as well as the severity of impairment. Jones et al. in 2009 enrolled 248 subjects with severe sepsis who were being treated with a standardized resuscitation protocol. The area under the ROC curve was 0.75 (95% CI 0.68-0.83) for SOFA score at time 0 and 0.84 (95%CI 0.77-0.90) at time 72 hours after presentation. [26] These findings show that the SOFA score demonstrates good accuracy at predicting in-hospital mortality early in the course of illness when screened in the ED.

### ED management

The initial management of the ED patient follows a simple and well-defined algorithm regardless of the etiology of the patient’s current illness. The ABCs of resuscitation instruct practitioners to evaluate the airway and, when required, establish a patent, definitive airway; evaluate for adequacy of breathing; and rapidly assess circulatory status. Emergency Department evaluation of critically ill patients also includes rapid establishment of adequate intravenous (IV) access (two 18 gauge or larger IVs at the outset of resuscitation), surveillance laboratory work, initiation of fluid bolus to reestablish preload if indicated, and obtaining appropriate diagnostic studies (Table1). With special focus on the severe sepsis patient, initial management should include blood and urine cultures, source control where appropriate, and rapid administration of appropriate antimicrobials. This is not to say that definitive identification of the site of infection and causative organism is required at the outset of care, but a focused physical examination generating an appropriate differential diagnosis of the infectious process is critical for delivering appropriate antimicrobials, establishing source control, and guiding hemodynamic resuscitation.

**Table 1:** First hours of management in life-threatening infections

**Time**	**Intervention**
**Triage**	·Screen for SIRS with vital signs
	·Screen for source by history and physical exam
	·Evaluate for organ dysfunction by assessing vital signs and level of consciousness
**Immediate**	·Assess ABCs
	·Establish definitive airway
	·Initiate NIPPVwhile preparing for intubation unless patient is apneic
	·Lung protective ventilator strategies
	·Obtain intravenous access (central or two peripheral)
	·Begin volume resuscitation
	·Avoid hyperoxia
**1st Hour**	·Send labs including lactate and blood cultures
	·Establish source control via broad spectrum antimicrobials and/or definitive management
	·Check ABG to ensure adequate gas exchange and avoid hyperoxia
	·Check plateau pressure to avoid barotrauma
	·Consider bedside ultrasound to assess cardiac function and IVC collapse
	·Order appropriate imaging
**Does Patient Qualify for EGDT?**	·SBP < 90 mmHg after 20-30 cc/kg bolus
	·Lactate > 4 mmol/L
**1st Two Hours**	·If EGDT eligible, place CVC in torso vein, assess CVP, ScvO_2_ ·If persistent hypotension (MAP < 65 mmHg), place arterial line
**Two Hours**	·Repeat lactate and calculate clearance
	·Assess total volume input and urine output
**Three Hours**	·Reassess input/output; assess resuscitation goals; is patient still volume responsive?
	·Repeat labs to assess for correction of organ dysfunction
**Four to Six Hours**	·Final disposition
	·If resuscitation goals met, enter maintenance phase
	·If not met, reassess
	·Consider corticosteroids for vasopressor dependent hypotension
	·Assess need for glucose control
**Every 20-30 Minutes**	·Serial reassessment of response to resuscitation

SIRS = systemic inflammatory response syndrome; ABC = Airway, Breathing, Circulation; IV = Intravenous; IVC = inferior vena cava; SBP = systolic blood pressure; EGDT = early goal directed therapy; CVC = central venous catheter; CVP = central venous pressure; MAP = mean arterial pressure; NIPPV = Noninvasive Positive Pressure Ventilation.

### Resuscitation: hemodynamic monitoring and physiologic optimization

Protocolized resuscitation of patients with severe sepsis and septic shock has become a widely recommended standard for patients with fluid refractory septic shock. Given the long ED boarding times in healthcare systems plagued by overcrowding, hemodynamic monitoring and physiologic optimization have become a necessary practice in acute care settings, prior to transfer to an ICU. Volume resuscitation (20-30 cc/kg of normal saline or lactated ringer’s solution over 15–30 minutes) should be initiated as soon as hypoperfusion is recognized regardless of patient location in the health care system. [9] In a randomized, controlled trial published in the *New England Journal of Medicine* in 2001, Rivers et al. demonstrated the time-critical nature of severe sepsis and septic shock, establishing the efficacy of a protocol for ED resuscitation known as Early Goal-Directed Therapy (EGDT). [6] The relevant hemodynamic end points for resuscitation employed in an algorithmic fashion in the EGDT arm of the trial were: a central venous pressure (CVP) of 8 to 12 mmHg; a mean arterial pressure (MAP) of 65 to 90 mmHg; and a central venous oxygen saturation (Scv0_2_) ≥ 70%. The standard therapy arm employed the following goals: a CVP of 8 to 12 mmHg; a MAP > 65 mmHg; and urine output ≥ 0.5 mL/kg/hr. The trial included 263 subjects randomized to the two arms and found a 16% absolute reduction in in-hospital mortality in the EGDT arm vs. the standard therapy arm. [6] This mortality benefit remained at 28 and 60 days.

In 2005 Otero et al. published a review analyzing the outcomes from 12 additional hospital EDs that had implemented an EGDT protocol. [27] The overall decline in mortality observed was from 44.8 ± 7.8% pre-implementation to 24.5 ± 5.5% post-implementation. These findings support the results of the initial randomized controlled trial by Rivers et al. from 2001. In the 2008 SSC Guidelines, the EGDT resuscitation algorithm was endorsed as a grade 1 C (strong recommendation, low quality of evidence) recommendation. However, several investigators have questioned the validity of the individual hemodynamic markers utilized in EGDT and continue to search for other less invasive mechanisms for assessing the progress and adequacy of resuscitation. [10,28].

As early as 1999 the Society for Critical Care Medicine issued a practice parameter for the management of severe sepsis in which one goal of early management was to correct the “early hypovolemic, hypodynamic phase of sepsis” with aggressive volume resuscitation. [8] Criticism has arisen as to whether this aggressive volume resuscitation could actually be harmful to patients with severe sepsis. In 2011, Boyd et al. performed a retrospective review of patients with vasopressor-dependent severe sepsis enrolled in the Vasopressin in Septic Shock Trial (VASST) and found that patients with a mean CVP of < 8 mmHg had the lowest rates of mortality followed by patients maintained at the guideline parameter of 8–12 mmHg. Finally, they noted that patients with a CVP > 12 mm Hg had the worst outcomes. [29] It is important to note that this study investigated the first four days of sepsis management, and that the earliest measurements documented were 12 hours after initiation of management, which may be a time frame that extends out of the early hypovolemic, hypodynamic phase of sepsis. This study actually questions the timing of when aggressive volume resuscitation should be decreased and not the role of aggressive fluid administration in the early phase of sepsis management. To further support this conclusion, Murphy et al. found that patients with septic shock and acute lung injury who underwent adequate initial fluid resuscitation and subsequent conservative late fluid management had better outcomes then patients who had only one or neither of these fluid management strategies. [30].

Central venous pressure is a controversial measurement in critical care management. Healthy individuals typically have CVP values ranging from 0 to 8 mmHg, while intubated patients will have higher CVPs due to the effects of positive pressure ventilation on intra-thoracic pressure. Marik et al. reviewed 24 studies examining the accuracy of CVP measurement. [28] They found that CVP is a very poor measurement of blood volume or of volume responsiveness; however, the studies reviewed were exclusively ICU or operating room studies and the patients enrolled may not be representative of severely septic ED patients at the most proximal phase of critical illness. The previously mentioned study by Boyd et al. found that the CVP measurements in the early phase of resuscitation correlated modestly with fluid balance at 12 hours but had no significant correlation on days 1–4. [29] This suggests that CVP may be most useful in the early, dynamic phase of severe sepsis and septic shock. In addition to its use as a resuscitation endpoint, Varpula and colleagues found that mean CVP the first 48 hours after admission to the ICU is independently associated with mortality in septic shock. [31]

Mean arterial pressure (MAP) is a macroscopic measure of global organ perfusion. It is the average pressure in one cardiac cycle and can be calculated using the following formulas: (1/3 *SBP) + (2/3* DBP) or DBP + (1/3* PP), where SBP is systolic blood pressure; DBP is diastolic blood pressure; and PP is pulse pressure. Mean arterial pressure, however, can be misleading when used as an endpoint of resuscitation in isolation because it is directly related to systemic vascular resistance (SVR) and cardiac output (CO). In compensated shock, a patient may have depressed CO masked by elevated SVR resulting in a normal or modestly elevated MAP. A MAP of ≥ 60 mmHg is needed to maintain nutrient and oxygen flow into tissue beds that may have altered autoregulation secondary to the sepsis syndrome or concomitantly administered medications. Although suggested MAP goals range from ≥ 60 to ≤ 90 mmHg [32,33] there are limited data that suggest using a MAP target ≥ 65 mmHg will result in increased cardiac output, improved microvascular function, and decreased blood lactate concentrations. [34].

Central venous oxygen saturation (ScvO_2_) is a measurement of the amount of oxygen remaining in the venous circulation after oxygen for cellular metabolism has been extracted systemically. In addition, abnormal ScvO_2_ values in patients who are hemodynamically stable can serve as a warning sign of impending sudden cardiovascular collapse^6^. It is measured via a central venous catheter with the tip placed at the atriocaval junction, a common procedure for the emergency medicine practitioner. Typically, the catheter is placed under ultrasound guidance in the right internal jugular vein. Other acceptable locations are the left internal jugular and subclavian veins. Values of ScvO_2_ can be obtained continuously via a specialized oximetric catheter or at the clinician’s discretion by drawing a venous blood gas from the distal port and measuring the oxygen saturation. The ScvO_2_ is often compared to an until recently more familiar measurement, the mixed venous oxygen saturation (SvO_2_) measured via a pulmonary artery catheter (PAC), occasionally placed by intensivists after the patient arrives in the ICU. Because of the added risk of placing a PAC, coupled with the infrequent use of this modality for hemodynamic monitoring, the ScvO_2_ is often utilized as a surrogate for the SvO_2_. Ladakis and colleagues found that in 61 critically ill ICU patients, the SvO_2_ and ScvO_2_ showed statistically significant correlation with an r-value of 0.945 (p < 0.03). [35] These findings were confirmed more recently in work published by Mozina and Podbregar who determined a Pearson’s correlation of 0.659 (p = 0.001) for the relationship between SvO_2_ and ScvO_2_. [36] These investigators did find that in patients with severe left heart failure there maybe a discrepancy in the correlation between these two measurements. The rationale behind the EGDT endpoint of an ScvO_2_ ≥ 70% is to ensure that oxygen delivery is sufficient to meet the oxygen demands of cellular metabolism at the tissue level. Investigators have also studied the clinical meaning of ScvO_2_ ≥ 90%, and whether this could be representative of poor oxygen extraction due to tissue deficits at the mitochondrial level. Pope and colleagues investigated over 600 patients from four academic ED EGDT registries and found that initial hyperoxia (defined as an ScvO_2_ ≥ 90%) was associated with a higher mortality rate (31 %, 95 % CI 21-48 %) when compared to initial normoxia, defined as an ScvO_2_ between 70 and 90% (23%, 95% CI 19-28 %)) and hypoxia, defined as an ScvO_2_ ≤70% (25%, 95% CI 20-31 %). [37] When investigating the maximum ScvO_2_obtained in the ED, both the hypoxia and hyperoxia groups exhibited a higher mortality rate, 40% and 34% respectively, vs. the normoxia group (21%). Further investigation is necessary to understand the cellular mechanisms for why the mortality in the hyperoxia group is similar to that in the hypoxia group and to determine plausible clinical interventions to correct hyperoxia during resuscitation.

Because of barriers to initiating invasive monitoring in many EDs, combined with the increased risk associated with placement of central venous access, investigators have continued to search for less invasive modalities for assessing hemodynamic status and outcomes of resuscitation. One value that has been investigated to assess adequacy of resuscitation is lactate clearance--examining change in serial lactate values over time. Nguyen and colleagues investigated the prognostic value of lactate clearance after six hours of ED resuscitation in a single center with an active EGDT program. Using a prospective observational convenience sample of severe sepsis and septic shock patients with a mean initial lactate of 6.9 mmol/L, they determined that a lactate clearance of ≥ 10% was associated with a significant reduction in mortality when compared to patients whose lactate clearance was < 10%. [38] Jones and colleagues attempted to investigate the non-inferiority of lactate clearance vs. ScvO_2_ monitoring as the final end-point of resuscitation in patients with severe sepsis or septic shock. [39] This study was a randomized controlled trial where one arm consisted of patients who were resuscitated to similar CVP, MAP and ScvO_2_ goals as used in the Rivers trial. The second arm targeted the same CVP and MAP goals; however, the third resuscitation goal was a lactate clearance of at least 10% when a repeat lactate was drawn two or more hours after the initial lactate was obtained. Researchers found that 23% of patients in the ScvO_2_ group died during hospitalization compared to 17% of patients in the lactate clearance arm (not statistically significant). This study was only designed as a non-inferiority trial; therefore, researchers concluded that there was no difference between outcomes in patients managed using lactate clearance vs. those managed by normalization of ScvO_2_. It is important to note that the initial lactate level in the lactate clearance group was 3.9 mmol/L vs. 4.2 mmol/L in the EGDT group while in Rivers *et al*’s trial the mean lactate in the EGDT group was 7.7 ± 4.7 mmol/L. [6] The difference in patient severity of illness is also reflected in the overall in-hospital mortality rates in both studies, 20% in Jones’s versus 38% in Rivers’ study, respectively. Although lactate clearance is an important resuscitation parameter, many patients in shock never generate abnormal lactate levels. [40] Furthermore, using a percent clearance may not be as useful as trending the absolute lactate level or normalization of the lactate level. As with all resuscitation parameters, lactate clearance is best used in conjunction with all other available data.

Another non-invasive tool to gauge the adequacy of resuscitation is measuring the decrease in inferior vena cava diameter during one respiratory cycle with bedside ultrasonography, known as the caval index. Nagdev and colleagues investigated the utility of correlating caval index with CVP as recorded by central venous catheterization. [41] Seventy-three patients with mixed critical illness were enrolled in this study and the researchers found that the correlation between caval index and CVP was −0.74 (95% CI −0.82 to −0.63). They also determined that the ability for a caval index ≥ 50% to predict a CVP of < 8 mmHg had a sensitivity of 91% (95% CI 71%-99%) and a specificity of 94% (95% CI 84%-99%). The utility of measuring caval index specifically in septic patients still needs to be further validated. Other limitations of ultrasound include inter-operator variability and variations in patient body habitus, which may alter the quality of images obtained.

Investigators have also examined the utility of impedance cardiography (ICG) as a non-invasive mechanism for measuring hemodynamic status in severe sepsis. Napoli et al. investigated the ability of cardiac index obtained in the ED using ICG monitoring to predict in-hospital mortality for patients who meet diagnostic criteria for EGDT. [42] The ICG device, a series of sensors to transmit current and measure impedance, placed on opposing sides of the neck and thorax, was used on 56 patients in this study. Investigators determined that the mean cardiac index in non-survivors was 2.3 L/min m^2^ (95% CI 1.6-3.0) and for survivors was 3.2 L/min m^2^ (95% CI 2.9-3.5). In their study, a cardiac index < 2 L/min m^2^ had a sensitivity of 43% (95% CI 18%-71%), a specificity of 93% (95% CI 80%-95%), a positive likelihood ratio of 5.9, and a negative likelihood ratio of 0.6 for predicting in-hospital mortality. This study indicates that non-invasive ICG monitoring may have some utility in predicting outcomes for patients undergoing EGDT, however, its utility in real-time hemodynamic monitoring needs to be further investigated.

Near infrared-spectroscopy has recently been used for non-invasive monitoring of tissue oxygen saturation (StO_2_). Investigators theorized that this measurement might correlate with ScVO_2_ measurements and could be utilized as a non-invasive surrogate for venous oxygen saturation. Napoli et al. investigated the relationship between these two measurements in 40 ED patients with severe sepsis or septic shock and found that StO_2_ systematically overestimated at lower ScvO_2_ and underestimated at higher ScvO_2_, with an overall fair correlation between the two measurements. [43] They concluded that the clinical use of StO_2_ in this patient population is unsubstantiated.

Several other dynamic endpoints of resuscitation use arterial waveform (including pulse pressure variability and stroke volume variability) or pulse oximetry waveform analysis. While they all show promise, they are unreliable in patients who are not intubated, who are intubated and being treated with low tidal volume ventilation, or who have cardiac dysrhythmias. Until further information is known and validated, the algorithmic approach to EGDT using invasive hemodynamic monitoring has proven success in the outcomes of patients with severe sepsis and septic shock and is still utilized and recommended by many in the emergency medicine community.

### Resuscitation: pharmacologic interventions

An additional factor within the algorithmic approach to hemodynamic resuscitation of the septic patient is pharmacologic intervention with vasopressors. Over the past decades, both dopamine and norepinephrine have been regarded as first line agents appropriate for the management of septic shock. In 2010, in *The New England Journal of Medicine,* De Backer and colleagues published a multicenter randomized controlled trial where patients with shock were assigned to receive either norepinephrine or dopamine as their initial vasopressor. [44] If patients were not responsive to titrated dosing of either medication, open label use of a second vasopressor was allowed. Investigators determined that there was a trend towards an increase in mortality in patients started on dopamine (52.5% vs. 48.5%, p = 0.10), and a higher rate of open label norepinephrine use after the initial dose of dopamine. In addition, investigators found that dopamine had a worse side effect profile than norepinephrine, with more arrhythmic events (24.1% vs. 12.4%, p < 0.001). Although no significant difference in mortality outcomes was noted, this study raises significant concerns regarding the safety of dopamine in the care of shock patients who are vasopressor-dependent.

Vasopressin has been utilized as an adjuvant therapy in the vasopressor-dependent septic shock patient. Prior studies have established a relative vasopressin deficiency in patients with septic shock, and investigators have theorized that vasopressin as low-dose hormone replacement may restore vascular tone and blood pressure, allowing for reduced doses of catecholamines. In a randomized double blind trial, Russell et al. assigned 778 septic patients who remained hypotensive despite norepinephrine therapy to receive either additional norepinephrine or low dose vasopressin. [45] Investigators found no difference in mortality between the vasopressin arm and the norepinephrine arm, and reported that there was no utility in adding vasopressin to the treatment algorithm of septic shock patients receiving norepinephrine.

During the last decade, tight glycemic control became one of the cornerstones of management of critically ill ICU patients. This emphasis on glycemic control stemmed from a 2001 study where Van den Berghe et al. addressed the issue of tight glucose control in a cohort of critically ill surgical patients. [46] The authors found that tight glycemic control reduced ICU mortality from 8.0% to 4.6% (p = 0.005) when compared to prior practice where insulin was only administered if the serum blood glucose was greater than 215 mg/dL. However, concerns about complications of hypoglycemia remained and investigators sought to address this question in a meta-analysis. A study, conducted by Wiener et al. analyzed 34 randomized controlled trials pertaining to tight glycemic control in the ICU. [47] They found that overall mortality rates did not differ between patients receiving tight glucose control vs. those who were cared for under a standard protocol (21.6% vs. 23.3%; RR 0.93; 95% CI 0.85-1.03). These studies were all conducted on patients being cared for in the ICU, and there are little data to guide the ED practitioner regarding glucose control during the first hours of the management of patients with life-threatening infections. It is reasonable for the emergency medicine provider to aim for a serum glucose of 8.3 mmol/l (150 mg/dl) and to treat any serum glucose over 12 mmol/l (~215 mg/dl).

The utility of steroid administration in the treatment of septic shock has been a significant topic of interest to investigators and clinicians. Annane et al. administered low dose hydrocortisone and fludrocortisone to patients with septic shock [48] and determined that, in patients with relative adrenal insufficiency, this regimen resulted in lower mortality and fewer days of vasopressor-dependency. These findings were utilized by practitioners until 2008 when the CORTICUS trial was published in *The New England Journal of Medicine*. CORTICUS was a multicenter, randomized, double-blind, placebo-controlled trial, in which patients were randomized to receive either 50 mg of hydrocortisone every 6 hours for five days or placebo, followed by a 6-day taper of either steroid or placebo. [49] The primary outcome was 28-day all cause mortality in patients who did not respond to a corticotropin stimulation test. Researchers found that there was no difference in mortality for patients receiving hydrocortisone vs. placebo, and therefore determined that there is no utility in treating patients with septic shock with steroids. Of note, the CORTICUS trial excluded all patients who had been administered long term steroids in the past 6 months or short term steroids in the last four weeks prior to enrollment while the prior study by Annane et al. did not exclude these patients. Another interesting comparison between these trials is that the CORTICUS trial enrolled patients within 72 hours of onset of shock, while the Annane et al. enrolled patients within three hours of onset of shock, raising concerns that these patients may be at varying stages within their disease processes and resuscitation. In 2010, investigators from the CORTICUS group published an additional analysis addressing the ability of hydrocortisone to protect against organ dysfunction in severe sepsis patients. Moreno et al. found that administration of hydrocortisone for 11 days vs. placebo resulted in improved performance on the SOFA score. [50] They found that hydrocortisone-treated patients had statistically significant improvement of cardiovascular dysfunction from day 0 to day 7 vs. those treated with placebo.

Packed red blood cell transfusion (PRBC) has been recommended for the anemic severely septic patient with inadequate oxygen delivery. The parameters often employed for transfusion are an ScvO_2_ < 70% with a hematocrit < 30%; however, there is significant skepticism that this intervention will truly affect the outcomes of patients with severe sepsis. Fuller et al. found that transfusion did not result in arrival at target ScvO_2_ of > 70%, nor did it result in less end organ dysfunction. Patients in this study were retrospectively analyzed, with one group receiving PRBC transfusion and the other undergoing resuscitation with purely crystalloid. [51] Further investigation is necessary to determine what role PRBC transfusion will have in sepsis resuscitation strategies, as PRBC transfusion has been associated with acute lung injury, infection, and increased mortality.

### Resuscitation: antimicrobial utilization

Appropriate antimicrobial administration and early, adequate source control are mainstays in the treatment of the septic ED patient. The importance of early antimicrobial therapy in patients with septic shock was brought to the forefront by Kumar et al. in 2006. Kumar and his colleagues completed a retrospective cohort study of 2,731 adult patients with septic shock, examining mortality in patients who received antimicrobials after the onset of recurrent or persistent hypotension. [5] They found that administration of an appropriate antimicrobial within one hour of identified hypotension resulted in a survival rate of 79.9%. Each hour’s delay in administration of antimicrobial resulted in a 7.6% decrease in survival.

Similarly, Gaieski et al. found overall mortality decreased by 13.7% in a cohort of 261 patients receiving uniform, algorithmic hemodynamic resuscitation when appropriate antibiotics were administered in less than one hour from triage time (33.2% vs. 19.5%; OR 0.3; 95% CI 0.11 to 0.83; p = 0.02). [52] Puskarich and colleagues further investigated this concept in post-hoc analysis of the LACTATES trial. [53] Researchers analyzed time from triage to first dose of antibiotics vs. time from shock recognition to first dose of antibiotics, and compared these data to in-hospital mortality. They found that 59% of patients received antibiotics after shock recognition and had an odds ratio of in-hospital mortality of 2.35 when compared with patients who had received antibiotics prior to shock recognition. This study also shows no increase in mortality associated with the delay in antibiotics over the first three hours after shock recognition. Of note is that Puskarich and colleagues did not analyze the “appropriateness” of antibiotic choice, and primarily looked at the first dose of antibiotics given, which may explain some difference in the two papers’ findings. Also, the overall mortality of patients in the LACTATES trial was significantly lower than that in Gaieski *et al*’s investigation. In summary, all of these papers endorse early antimicrobials as a component of a comprehensive resuscitation protocol, with data to support the use of appropriate antimicrobial choice in addition to administration prior to shock recognition. The combination of an antimicrobial algorithm coupled with an aggressive resuscitative protocol has resulted in improved outcomes for patients with severe sepsis and septic shock.

### Care bundles

With significant advancement in the care of the septic patient and implementation of guidelines for bundled care, investigators involved in the Surviving Sepsis Campaign demonstrated a decline in 28 day mortality from 37% to 30.8% over two years. [13] Compliance with the initial six hour bundle, termed the resuscitation bundle, increased from 10.9% to 31.3% of subjects over the two years after initiation of the SSC guidelines. It is difficult to interpret if the improved outcomes that were observed should be attributed to the SSC Guidelines, or to a heightened appreciation by emergency medicine and critical care practitioners that time sensitive care of sepsis begins in the ED.

In their study published in 2007, the Finnsepsis study group performed a prospective observational study of Finnish patients admitted to over 82% of all multidisciplinary ICUs in Finland. They found that compliance with sepsis management bundle guidelines in patients with severe sepsis was poor. In fact, no female patient (whose tidal volumes per predicted body weight could be calculated) was ventilated with a tidal volume of less than 6 ml/kg. Despite this, ICU and hospital mortality rates (15.5% and 28.3%, respectively) were lower than in many other published studies. [54] The most important predictor of survival was administration of antimicrobials within three hours of presentation.

With the refinement of diverse methodologies for identifying and treating patients with severe sepsis or septic shock at the most proximal point of entry into the healthcare system, resuscitation is beginning earlier and patient outcomes seem to be improving. Bundled care of the septic patient prompts providers to employ endpoints of resuscitation and emphasizes time-sensitive critical interventions such as early antimicrobial administration and aggressive volume resuscitation in the golden hours of sepsis prior to the development of multi-organ compromise. More research is necessary to confirm that use of bundles indeed directly leads to improved outcomes.

## Conclusion

In summary, severe sepsis and septic shock are medical emergencies that affect hundreds of thousands of individuals annually and result in death in about one third of these patients. Severe sepsis and septic shock are disease processes that require aggressive, time sensitive interventions, which has lead to the adoption of the practice of early-goal directed therapy and other aspects of bundled sepsis care in emergency departments internationally (Table2). Since the inception of this concept of early aggressive management of the septic patient, many advances have occurred to help further the care of these patients. Advances in methods for hemodynamic monitoring, the use of appropriate antimicrobial therapy, more sophisticated understanding of the risks and benefits of aggressively controlling glucose levels, choice of vasopressor therapy, and the utility of steroid implementation in the septic patient have lead to significant improvement in patient care. Despite controversy with regard to implementation and utilization of the Surviving Sepsis Campaign recommendations, emergency departments that have been compliant with the guidelines have seen significant improvement in their outcomes and more survivors. In summary, severe sepsis is a time critical disease, and therefore, one of significant concern to the emergency medicine physician.

**Table 2:** Common Causes of Severe Sepsis by Organ System*

**Organ**	**Etiology**	**Diagnostic Study**
**Lungs**	Pneumonia	Chest radiograph
	-Hospital acquired	CT scan of the chest
	-Community acquired	
**Abdomen**	Appendicitis	Abdominal radiograph
	Cholecystitis/Cholangitis	CT scan of the abdomen and pelvis
	Bowel perforation	Right upper quadrant abdominal ultrasound
	Peritonitis	HIDA scan
**Urinary Tract**	Cystitis	Urine analysis and culture
	Pyelonephritis	Renal ultrasound
	Urosepsis	*Remove any instrumentation ie. Indwelling foley catheter
**Skin**	Cellulitis	Isolation and culture
	Wound	
**Bones**	Osteomyelitis	Bone scan or MRI
	Sinusitis	CT scan of the maxillofacial bones and sinuses
**Central Nervous System**	Meningitis/Encephalitis	Lumbar puncture
	Spinal abscess	MRI of the brain and/or spine
		CT scan of the brain
**Blood**	Secondary from other source	Blood culture
	Catheter associated	*Remove any instruments that may seed infection ie. central line, pacemaker wires
**Unknown**	*Approximately 20% of patients will have an unknown source.	

*Adapted from Levy et al. The Surviving Sepsis Campaign: results of an international guideline-based performance improvement program targeting severe sepsis. *Crit. Care Med.* 2010;38(2):367–374.

## Competing interests

The author(s) declare that they have no competing interests.

## Authors’ contributions

SMP, MG, and DFG created the outline and conducted the literature search; SMP wrote the first draft of the manuscript; MG and DFG edited the first draft and added additional references; DFG created Table 1; SMP created Table 2; all authors read and approved the final manuscript.
